# Prognostic factors of nasopharyngeal carcinoma patients in a tertiary referral hospital: a retrospective cohort study

**DOI:** 10.1186/s13104-017-2990-1

**Published:** 2017-12-06

**Authors:** Ab Hamid Siti-Azrin, Bachok Norsa’adah, Nyi Nyi Naing

**Affiliations:** Unit Biostatistics and Research Methodology, Universiti Sains Malaysia, 16150 Kubang Kerian, Kelantan, Malaysia

**Keywords:** Nasopharyngeal carcinoma, NPC, Prognostic factors, Survival, Age, Metastases, Stage

## Abstract

**Background:**

Nasopharyngeal carcinoma (NPC) exhibits a distinctive racial and geographic distribution. Many studies have reported varied significant prognostic factors affect the survival of NPC patients. Hence, this current study aimed to identify the prognostic factors of NPC patients registered in a tertiary referral hospital.

**Methods:**

The records of one hundred and thirty-four NPC cases confirmed by histopathology in Hospital Universiti Sains Malaysia (USM) between 1st January 1998 and 31st December 2007 that fulfilled the inclusion and exclusion criteria were retrospectively reviewed. Simple and multiple Cox proportional hazard regression analyses were performed to determine the significant prognostic factors affect the survival of NPC patients.

**Results:**

The mean (SD) age of patients diagnosed with NPC was 48.12 (15.88) years with Malay was the largest ethnic group compared to other ethnicities. Most of patients had locally advanced stage IV (40.6%) and stage III (39.1%) of NPC. The overall median survival time of NPC patients was 31.30 months (95% CI 23.76, 38.84). The significant prognostic factors that influenced the survival of NPC patients were older age (HR 1.03, 95% CI 1.01, 1.04), metastases (HR 2.52, 95% CI 1.01, 6.28) and stage IV disease (HR 4.50, 95% CI 1.66, 9.88).

**Conclusion:**

Older age, the presence of metastases and late stage are significant prognostic factors that influence the survival of NPC. Therefore, it is important to provide education to public and to raise awareness to diagnose NPC at an earlier stage and before the presence of metastases.

## Background

Nasopharyngeal carcinoma (NPC) exhibits a distinctive racial and geographic distribution; NPC is more prevalent in Southern China and Southeast Asia [1]. Based on the global cancer statistics from the International Agency for Research on Cancer, there were an estimated 84,400 new NPC cases and 51,600 deaths in 2008, representing approximately 0.7% of the global cancer burden [1]. In Malaysia, NPC ranked the fourth most common cancer among the entire population and the third most common cancer among males [2]. Based on the cancer incidence in Peninsular Malaysia from 2003 to 2005, 900 cases of NPC were registered with the National Cancer Registry [2].

The early detection of NPC is important because patients with early disease have a significantly higher chance of being cured and can be spared the financial burden and associated toxicities of additional chemotherapy. Many NPC patients presented with an advanced stage of the disease [3]. Licitra et al. [4] explained that nasopharyngeal tumours initially develop without producing any signs and symptoms as a result of location and the anatomical structure of the nasopharynx. NPC showed the highest propensity for lymphatic spread and distant metastases. Over 70% of NPC patients have neck masses of 6–15% and present with synchronous distant metastases at the initial diagnosis [5]. The most common sites of metastases are the bones, lungs and liver [6].

The current study was the extension from the previous study published in the year 2014 [7]. The cohort for both studies was the same which involve retrospective cohort study of 134 NPC patients. However, these two studies were distinguished by their objective. The previous study aimed to determine the median survival time of NPC patients. The survival time of NPC patients were estimated by univariable analyses which was Kaplan–Meier survival analysis. While, the current study aimed to identify the prognostic factors of NPC patients. The analysis involves the multivariable analyses. Simple and multiple Cox proportional hazard regression analyses were performed to identify the significant prognostic factors that influence the survival of NPC patients.

Various studies of the prognostic factors of NPC have been published, but few have been conducted in Malaysia. Knowledge about prognostic factors may help to recognize patients who are at risk and therefore facilitate treatment decisions, preventive strategies, education and counselling. The prognostic factors of NPC can be divided into patient-related (age, gender and ethnicity), disease-related (histology type, TNM classification and staging) and treatment-related factors. Different studies have reported various significant prognostic factors affect the survival of NPC patients. Thus, this study was conducted to identify the prognostic factors that influence the survival of NPC patients in a tertiary referral hospital.

## Methods

This is a retrospective cohort study that involved a retrospective record review of 134 newly diagnosed NPC patients, who were histologically confirmed to have NPC in Hospital USM between 1^st^ January 1998 and 31st December 2007. Patients with more than 30% incomplete data and an indefinite date of diagnosis, date last seen or date of death were excluded from the study. The sample size was calculated using PS software with the following parameters: significance level, α = 0.05 (two tailed); power, 1 − β = 0.8; accrual time during which patients were recruited, A = 120 and additional follow up after the end of recruitment, F = 12. An additional 20% was added, based on the estimated 20% of data missing due to loss to follow up. An estimated 178 samples were required to adequately rejecting the null hypothesis. No sampling method was applied for this study.

Information on the patients’ living status was obtained from the record and was confirmed with the National Registration Department. Patients were considered dead if their deaths were caused by NPC and its complications within the study period, whereas patients were considered as censored if they survived beyond the end of the study period or those whose status could not be determined at the end of study. Only single researcher retrieved the needed information.

The study had ethical approval from the Human Research Ethics Committee of USM. Informed written consent was not applicable because this study only involved a retrospective record review of NPC patients. Permission to access patient’ folders or records was obtained from the Hospital Director of Hospital USM. A confidential code was used in the data collection sheet to represent each patient.

## Statistical analyses

Data analysis was conducted using SPSS version 20 [8] and STATA software, version 11 [9]. The survival time was measured from the date of NPC diagnosis to death. Simple and multiple Cox proportional hazard regression analyses were performed to identify the significant prognostic factors that influence the survival of NPC patients. Crude and adjusted hazard ratios (HRs) and 95% confidence intervals (CIs), Wald statistics and corresponding *p* values were reported. The level of significance, α, was set at 0.05 (two tailed).

## Results

NPC patient characteristics are shown in Table 1. The mean (SD) age at diagnosis was 48.12 years (15.88) [7]. Only 5.2% of the sample was paediatric patients. Only 24.6% of patients had co-morbidities at the time of diagnosis [7]. Most of these patients presented with symptoms of neck swelling (73.1%) at diagnosis. WHO type III constituted approximately 69.4% of all histological types [7]. The majority of patients presented with T4 (48.1%), N3 (32.3%) and no metastases (82.7%) [7]. A majority of the patients had locally advanced NPC: stage IV (40.6%) and stage III (39.1%). Most of the patients (58.2%) received combination radiotherapy and chemotherapy, 28.4% of patients were treated with radiotherapy alone and 5.2% of patients were treated with chemotherapy alone. The overall median survival time of NPC patients was 31.30 months (95% CI 23.76, 38.84).

**Table 1:** Characteristics of NPC patients in Hospital USM (n = 134)

Clinical characteristics	Died frequency (%)	Censored frequency (%)	Total frequency (%)
Gender			
Female	18 (56.2)	14 (43.8)	32 (23.9)
Male	62 (60.8)	40 (39.2)	102 (76.1)
Ethnicity			
Malay	66 (60.6)	43 (39.4)	109 (81.3)
Chinese	13 (54.2)	11 (45.8)	24 (17.9)
Other	1 (100.0)	0 (0.0)	1 (0.7)
T-classification			
T1	6 (46.2)	7 (53.8)	13 (9.7)
T2	8 (50.0)	8 (50.0)	16 (11.9)
T3	15 (62.5)	9 (37.5)	24 (17.9)
T4	38 (59.4)	26 (40.6)	64 (47.8)
N-classification			
N0	8 (53.3)	7 (46.7)	15 (11.2)
N1	11 (50.0)	11 (50.0)	22 (16.4)
N2	18 (48.6)	19 (51.4)	37 (27.6)
N3	30 (69.8)	13 (30.2)	43 (32.1)

## Prognostic factors

The analysed prognostic factors of NPC were patient-related (age, gender and ethnicity), disease-related (histology type, TNM classification and staging) and treatment-related. Univariate analysis (simple Cox regression analysis) revealed that five variables were statistically significant as prognostic factors: age, cranial nerve palsies, metastases, staging and treatment (Tables 2, 3, 4, 5).

**Table 2:** Prognostic factors of NPC patients in Hospital USM using simple Cox regression based on socio-demographic (n = 134)

Socio-demographics	b (SE)	Crude HR (95% CI)	Wald statistic	*p* value
Age (year)	0.02 (0.01)	1.02 (1.01, 1.03)	8.01	0.005
Gender				
Female	0.00	1.00	–	–
Male	0.24 (0.27)	1.28 (0.75, 2.16)	0.81	0.368
Ethnicity				
Malay	0.00	1.00	–	–
Chinese	− 0.23 (0.30)	0.80 (0.44, 1.45)	0.55	0.457
Other	− 0.09 (1.01)	0.92 (0.13, 6.63)	0.01	0.930
Co-morbidities				
No	0.00	1.00	–	–
Yes	− 0.23 (0.27)	0.78 (0.46, 1.35)	0.73	0.393

**Table 3:** Prognostic factors of NPC patients in Hospital USM using simple Cox regression based on symptoms (n = 134)

Symptoms (presence)	b (SE)	Crude HR (95% CI)	Wald statistic	*p* value
Neck swelling	0.52 (0.27)	1.69 (0.99, 2.88)	3.63	0.057
Nasal presentation	0.04 (0.23)	1.05 (0.67, 1.64)	0.04	0.847
Nasal blockage	− 0.10 (0.20)	0.90 (0.57, 1.42)	0.20	0.655
Nasal discharge	− 0.50 (0.40)	0.61 (0.28, 1.33)	1.56	0.212
Epistaxis	− 0.09 (0.23)	0.92 (0.59, 1.43)	0.15	0.699
Aural presentation	0.25 (0.23)	1.29 (0.83, 2.00)	1.27	0.260
Tinnitus	0.02 (0.28)	1.02 (0.59, 1.76)	0.00	0.949
Hearing loss	0.29 (0.23)	1.33 (0.85, 2.10)	1.55	0.213
Deafness	0.80 (0.72)	2.23 (0.54, 9.16)	1.23	0.267
Serous otitis	− 1.23 (0.72)	0.29 (0.07, 1.19)	2.94	0.086
Cranial nerve palsies	0.56 (0.22)	1.74 (1.12, 2.71)	6.16	0.013
Opthalmegia	0.00 (0.37)	1.00 (0.48, 2.08)	0.00	0.997
Visual changes	0.49 (0.26)	1.64 (0.99, 2.72)	3.64	0.056
Numbness	− 0.07 (0.46)	0.94 (0.38, 2.31)	0.02	0.886
Hoarseness of voice	0.26 (0.51)	1.30 (0.47, 3.55)	0.25	0.615
Headache	0.17 (0.24)	1.19 (0.74, 1.92)	0.51	0.474

**Table 4:** Prognostic factors of NPC patients in Hospital USM using simple Cox regression based on clinical characteristics (n = 134)

Clinical characteristics	b (SE)	Crude HR (95% CI)	Wald statistic	*p* value
Histologic type				
WHO type I	0.00	1.00	–	–
WHO type I	0.37 (0.25)	1.45 (0.892, 2.37)	2.25	0.134
WHO type I	0.05 (0.59)	1.06 (0.33, 3.38)	0.59	0.928
T-classification				
T1	0.00	1.00	–	–
T2	0.15 (0.54)	1.16 (0.40, 3.36)	0.08	0.780
T3	0.45 (0.49)	1.56 (0.60, 4.05)	0.85	0.357
T4	0.40 (0.44)	1.49 (0.63, 3.52)	0.81	0.368
N-classification				
N0	0.00	1.00	–	–
N1	− 0.03 (0.47)	0.97 (0.39, 2.42)	0.00	0.952
N2	0.25 (0.43)	1,28 (0.55, 2.95)	0.33	0.567
N3	0.77 (0.40)	2.16 (0.99, 4.73)	3.69	0.055
Metastases				
M0	0.00	1.00	–	–
M1	1.11 (0.44)	3.02 (1.29, 7.09)	6.45	0.011
Staging				
I–II	0.00	1.00	–	–
III	0.45	1.81 (0.75, 4.38)	1.72	0.190
IV	0.44	3.41 (1.44, 8.08)	7.79	0.005

**Table 5:** Prognostic factors of NPC patients in Hospital USM using simple Cox regression based on treatment (n = 134)

Treatment	b (SE)	Crude HR (95% CI)	Wald statistic	*p* value
Radiotherapy		1.00	–	–
Chemotherapy	2.12 (0.49)	8.37 (3.18, 22.01)	18.54	< 0.001
Combination radiotherapy and chemotherapy	− 0.13 (0.26)	0.88 (0.53, 1.46)	0.26	0.613

A 1-year increase in age increased the risk of dying due to NPC or its complications by 1.02-fold (95% CI 1.01, 1.03). Those NPC patients who had cranial nerve palsies at the time of diagnosis had a 1.74-fold (95% CI 1.12, 2.71) higher risk of death compared with those without cranial nerve palsies. Those NPC patients who had metastases at the time of diagnosis had a 3.02-fold (95% CI 1.29, 7.09) higher risk of death compared with those without metastases. Those patients who were diagnosed with stage IV NPC had a 3.41-fold (95% CI 1.44, 8.08) higher risk of death compared with those diagnosed with stage I-II NPC. Those NPC patients who received chemotherapy had an 8.37-fold (95% CI 3.18, 22.01) higher risk of death compared with those who received radiotherapy.

Insignificant factors were included in multiple cox regression analyses because the factors were clinically important on the survival of NPC patients. Factors include age, gender, ethnicity, co-morbidities, histology type, TNM classification, staging and treatment-related factors. The multivariate Cox analysis found that older age, metastases and stage IV NPC were independent prognostic factors for the survival of NPC patients in Hospital USM (Table 6).

**Table 6:** The final simple and multivariate Cox proportional hazard regression model of prognostic factors associated with the survival of NPC patients in Hospital USM (n = 134)

Variables	Simple Cox regression			Multiple Cox regression		
	b	Crude HR (95% CI)	*p* value	b	Adjusted HR (95% CI)	*p* value
Age (year)	0.02	1.02 (1.01, 1.04)	0.005	0.03	1.03 (1.01, 1.04)	0.001
Metastases						
No	0.00	1.00	–	0.00	1.00	–
Yes	1.11	3.02 (1.29, 7.09)	0.011	0.92	2.52 (1.01, 6.28)	0.048
AJCC stage						
I–II	0.00	1.00	–	0.00	1.00	–
III	0.45	1.81 (0.75, 4.38)	0.190	0.88	2.42 (0.98, 5.99)	0.056
IV	0.44	3.41 (1.44, 8.08)	0.005	1.40	4.05 (1.66, 9.88)	0.002

A backward stepwise Cox proportional hazards regression model was applied. Two-way interaction and multicollinearity were assessed and not found. A log-minus-log plot, a hazard function plot and Schoenfeld residuals were applied to test the model assumptions. Martingale residuals, Cox-Snell residuals, deviance residuals and influence analysis were used to assess the fit of the model and to identify influential cases

*b* regression coefficient

## Discussion

In the current study, the age of the patients ranged between 11 and 93 years old. The mean age at diagnosis of the NPC patients was 48.12 years old (SD 15.88). This finding was comparable to the mean age reported by El-Sherbieny et al. who found a median age of 48 years old and a range of 14–78 years old [10]. A 35-year study in Hong Kong (year 1983 and 2008) reported that the incidence of NPC increased over the age, peaking at ages 55–59 years old and showing a decline thereafter [11]. This indicates that NPC occurred mostly among adolescent and adult age groups.

In the current study, the age at diagnosis was found to have an impact on the survival of NPC patients. Most previous studies have also stated that the age at diagnosis had a significant influence on the risk of dying for NPC patients [12, 13]. However, El-Shierbieny et al. [12] reported that age was not a significant prognostic factor [10]. Younger patients generally have a well performance status and less co-morbidity, which may add to better tolerance of radiotherapy or chemotherapy, thereby resulting in better survival. The vast majority of previous studies employed conventional radiotherapy technology. The radiotherapy technique has an impact on the dose delivered to the local lesion and could pose a tolerance problem, thereby affecting tumour control and survival in patients [12]. Unlike intensity-modulated radiotherapy, it offers the potential for improved treatment outcomes because patients, including older patients, have a high tolerance for the therapy [12].

The other significant prognostic factor that affected the survival of NPC patients in the current study was the presence of metastases. Only 17.3% of the patients in the current study presented metastases. Wang et al. reported that distant metastasis was a significant prognostic factor [14]. Similarly, Liu et al. [15] also reported that metastases influence the survival of NPC patients. In the current study, the authors compared patients who had metastases at the time of diagnosis and after receiving primary radiotherapy [15]. Other factors, such as age and the site of metastasis were considered. The majority of NPC patients in the current study who had metastases were older. A previous study did discuss the age of the patients diagnosed with metastases because older patients have shorter survival times than younger patients.

Staging was a significant prognostic factor in the current study, mostly when comparing stage I–II with stage IV. The result was similar to that reported by other studies [16]. Most of NPC patients present with an advanced stage of the disease [3]. In the current study, 79.7% patients were diagnosed at advanced stage III and IV. The survival of NPC patients decreased as the stage of disease increased [16]. The factors that contributed to late presentation in the current study remain unclear. Possible factors in the late presentation of NPC include a delay in seeking medical advice, the confusing nature of the presented symptoms, which can be misleading to the clinician, the difficult nature of a clinical examination of the nasopharynx and the spread of a silent submucosal lesion with a normal appearance during examination of the nasopharynx [17]. Licitra et al. [4] explained that NPCs initially grow without producing signs and symptoms due to the location and the anatomical structure of the nasopharynx.

A study by Sing and Subramaniam on late presentation showed a 176-day mean delay between presenting with symptoms and seeking professional attention [18]. The major reasons for this phenomenon were that patients were unaware of NPC and its seriousness (72%), had no pain (30%) and sought out traditional treatment first (24%) [18]. The delay was particularly acute with patients presenting with ear symptoms (266 days), followed by those presenting with neck swelling (94 days) [19]. Therefore, it is importance to increase public education either in the general public or by general practitioners to improve the pickup rate during earlier stages of the disease [20]. Early detection of NPC was believed to decrease the mortality. People should consult a doctor early when experiencing any symptoms of NPC. Education regarding the symptoms and signs of NPC should be conducted to the public.

The treatment modality was not a significant prognostic factor in the current study. A majority of the patients (58.2%) received combination radiotherapy and chemotherapy and 28.4% of patients were treated with radiotherapy alone. The 5-year survival rate of the patients received combination radiotherapy and chemotherapy were 44.6 and 38.6% for patients were treated with radiotherapy alone. The 5-year survival rate of the patients received chemotherapy alone was undetermined. Those NPC patients who received chemotherapy alone had an 8.37-fold higher risk of death compared with those who received radiotherapy alone. The patients treated with chemotherapy alone had a higher stage as compared to those received radiotherapies alone. Due to the restricted number of patients who completed treatment, the effect of the treatment modalities might be biased. Of the total, 44% of NPC patients in the current study did not attend the follow-up appointment.

Radiotherapy is the suggested treatment for non-metastatic disease, due to its complex anatomic location and high radio-sensitivity [21]. Radiotherapy has a high cure rate for patients in the early stages, whereas chemotherapy is the treatment of choice for advanced stage disease. Studies have shown that concurrent chemo-radiotherapy is the most effective treatment. In the current study, higher 5-year survival was found in patients treated with combination radiotherapy and chemotherapy compared with radiotherapy alone; this difference was significant. Other studies also revealed that patients who underwent concurrent chemo-radiation treatment had better survival compared with those who received radiotherapy [22, 23]. All of these studies compared patients who received concurrent chemo-radiation with radiotherapy alone. A study by Zhang et al. showed that the survival of NPC patient was almost 100%, whereas Chan et al. showed that the survival was 70% [24, 25]. Most of the patients diagnosed at an early stage underwent chemo-radiation, and thus, a longer survival time was noted in this group.

The major limitation of the current study was the use of secondary data. Several patients’ records could not be found; since the patients did not attend for follow-up over a prolonged time. In addition, some information in the records such as blood results, histopathology and computerised tomography scan reports was incomplete or lost. Some reports were not clear and were confusing because different doctors reported data in different ways. All of the missing, unavailable, incomplete and ambiguous data could be avoided if the information was recorded properly by following standard criteria. Thus, the factors were omitted from the model. Approximately 24 NPC patients had incomplete data for multiple factors were excluded from the analysis. The excluded cases might have different survival times, resulting in an under- or overestimation of our outcome. Unfortunately, the authors unable to provide the information on the prognostic value of plasma Epstein–Barr virus DNA for advanced NPC since it not routinely tested for all the NPC patients in the institution.

The pre-determined power of the current study was 80%. However, the priori calculated sample size was not achieved in the current study due to small number of NPC cases available in the institution and inadequate recording of the medical record. The authors recalculated post hoc power of the study; which was 77.9%. Obviously, this was one of the current study’s limitations. Further study with a larger sample size should be done to determine more significant prognostic factors affect the survival of NPC patients. Expanding the research setting (i.e., multi-centre research) can hold large sample size, enlarge the coverage and guarantee the validity of the study; this expansion would include the study of a larger sample size and more factors related to NPC. Prospective cohort or interview studies can be used to study NPC patients were this designs can have least missing or incomplete data.

The paediatric patients had a lower risk for mortality relative to adults. So, further study should be done on the survival rate, survival time, prognostic factors and late sequelae of childhood nasopharyngeal carcinoma.

## Conclusion

The significant prognostic factors that influence the survival of NPC patients in Hospital USM were older age, the presence of metastases and stage IV disease. Attempts to increase early diagnoses must be founded. It is crucial to provide public education and increase the awareness of this highly prevalent cancer.

## Abbreviations

NPC: nasopharyngeal carcinoma
USM: Universiti Sains Malaysia
SD: standard deviation
HR: hazard ratio
CI: confidence interval
T: tumour
N: nodes
AJCC: American Joint Committee on Cancer
b: regression coefficient
